# Do uncemented humeral components perform better than cemented humeral components in reverse total shoulder arthroplasty for acute proximal humerus fracture? A New Zealand Joint Registry study

**DOI:** 10.1016/j.jseint.2025.10.009

**Published:** 2025-10-30

**Authors:** Alex B. Boyle, Scott M. Bolam, Chris M.A. Frampton, Peter Poon, Adam Dalgleish, Ryan Gao

**Affiliations:** a Department of Orthopaedic Surgery, Auckland City Hospital, Auckland, New Zealand; b Department of Orthopaedic Surgery, North Shore Hospital, Auckland, New Zealand; c Department of Surgery, The University of Auckland, Auckland, New Zealand; d Department of Medicine, The University of Otago, Christchurch, New Zealand

**Keywords:** Reverse total shoulder arthroplasty, Proximal humerus fracture, Trauma, Cemented, Uncemented

## Abstract

**Background:**

Reverse total shoulder arthroplasty (rTSA) is increasingly used in unreconstructible and comminuted proximal humerus fractures. There is ambiguity as to whether uncemented or cemented humeral components (stems) have better survival and patient-reported outcome measures in this context. The aim of this study was to compare implant survival, risk of revision, reason for revision, and functional outcomes between cemented and uncemented stems for rTSA performed for acute proximal humerus fractures.

**Methods:**

Prospectively collected data from the New Zealand Joint Registry, a national database with capture >95%, were used to compare the survival rates and Oxford Shoulder Scores of rTSA performed for trauma (trauma rTSA) using cemented or uncemented stems between January 2002 and December 2024. Reason for revision and patient demographics were recorded. Revision rates (rates per 100 observed component years) and functional outcomes (Oxford Shoulder Score at 6-month and 5-year follow-up) were compared using a multivariate Cox proportional hazards regression model and adjusted by age, sex, American Society of Anesthesiologists class, and surgeon volume of rTSA per year.

**Results:**

Over the 22-year study period, 843 rTSA procedures were performed for acute proximal humeral fracture representing 4,668 component years. Of these trauma rTSA procedures, 326 utilized a cemented stem (cemented trauma rTSA) and 517 utilized an uncemented stem (uncemented trauma rTSA). The number of revisions per 100 component-years for cemented trauma rTSA was 0.64, compared to 0.36 for uncemented trauma rTSA. This difference was not statistically significant (*P* = .122). Mean Oxford Scores 6 months postoperatively were 30.4 for cemented trauma rTSA and 31.3 for uncemented trauma rTSA (*P* = .365). Mean Oxford Scores 5 years postoperatively were 36.2 for cemented trauma rTSA and 39.5 and for uncemented trauma rTSA (*P* = .049), although this is less than the minimally clinically important difference.

**Conclusion:**

In the context of increasing use of rTSA for acute proximal humerus fractures, revision rates and patient-reported outcomes are similar between cemented and uncemented humeral stems. Uncemented humeral components are therefore an acceptable first-line treatment for proximal humerus fractures in appropriate patients.

Proximal humerus fractures have an increasing incidence in the context of an aging global population.^9^ Despite an overall decrease in the proportion of these fractures managed operatively, reverse total shoulder arthroplasty (rTSA) for acute fracture has increased significantly in recent years^14^; Increased use of rTSA in proximal humerus fractures is in the context of a broader utilization of rTSA,^3^ which now comprises a significant majority of shoulder arthroplasty performed in New Zealand,^27^ Australia,^28^ the United Kingdom,^29^ and the United States.^1^

With increasing use of rTSA for proximal humerus fractures, increased scrutiny is being placed on the choice of humeral component, in particular the choice of a cemented or uncemented stem.^21^ The osteoporotic bone typically seen in proximal humerus fracture patients^23^^,^^24^ is associated with periprosthetic fractures, subsequent fragility fractures, and all-cause revision arthroplasty.^5^ Cemented implants have theoretical advantages in terms of creating a bone cement interface in osteoporotic bone and have therefore previously been considered the gold standard for these patients.^5^^,^^13^ However, cement brings with it potential issues including increased operative time, cost, cement disease, and further considerations in revision surgery.^2^^,^^7^^,^^25^^,^^26^ Because of this, uncemented stems are being increasingly utilized^27^ but concerns have been raised about their use in the fracture population due to the frequent disruption of the metaphyseal region, which may lead to reliance on diaphyseal fixation alone.^7^^,^^16^^,^^20^ For both stem types, loosening is a key concern.

With these complex considerations at play, it remains unclear whether cemented or uncemented humeral components are most appropriate in the context of rTSA for acute proximal humerus fractures. The aim of this study was to compare implant survivorship, patient-reported outcome measures, and reason for revision for patients who had undergone cemented rTSA or uncemented rTSA for proximal humerus fracture, using data from the New Zealand Joint Registry.

## Materials and methods

All patients who underwent primary rTSA in New Zealand between January 2002 and December 2024 were identified in the New Zealand Joint Registry (NZJR), a national database which capture >95% of arthroplasty procedures.^18^ Of these patients, those who had rTSA performed for the primary indication of ‘trauma’ were identified for a total of 843 rTSA procedures (trauma rTSA procedures). Indication for surgery is selected by the primary surgeon at time of surgery, who must complete a form immediately postoperatively. These trauma rTSA procedures were then divided into 2 groups; those performed with a cemented stem (cemented rTSA) and those performed with an uncemented stem (uncemented rTSA).

The NZJR is linked to the New Zealand Registry of Birth, Deaths, and Marriages every six months to ensure accurate data capture on patient mortality. To record postoperative patient outcomes, the Oxford Shoulder Score (OSS)^6^ is sent to all patients at six months postoperatively and then every subsequent five years. All patients registered in the NZJR have consented to the collection and the analysis was exempt from a separate ethical approval process as per NZJR policy.

The primary outcomes measures were revision-free implant survival and postoperative functional outcomes (OSS at six months, five years, and ten years). The secondary outcome measure was the reason(s) for revision. A standard definition of revision was used: an operation in which 1 or more components are exchanged, removed, manipulated, or added. The NZJR allows operating surgeons to record more than 1 reason for revision.

### Statistical analysis

The cemented trauma rTSA and uncemented trauma rTSA groups were analyzed. Baseline differences between the groups with regards to patient characteristics (age, gender, American Society of Anesthesiologists grade^22^) and operative characteristics (surgeon volume) were compared using one-way analysis of variance with post hoc Tukey test or Chi-Square tests. Revision rates per 100 observed component years were calculated as the total number of revisions divided by the total number of component years multiplied by 100. Use of rates per 100 observed component years controls for the variability in follow-up time within the large group of patients within the NZJR. Multivariate Cox proportional hazards regression models were used to compare the revision rates in the 3 groups and were adjusted based on patient age at surgery, American Society of Anesthesiologists score, surgical indication, sex, and surgeon volume of rTSA per year. *P* values below 0.05 were considered significant in all cases.

## Results

### Patient demographics

Over the 22-year study period, 843 rTSA procedures were performed in the context of acute proximal humeral fracture representing 4,668 component years. Of these trauma rTSA procedures, 326 utilized a cemented stem (cemented trauma rTSA) and 517 utilized an uncemented stem (uncemented trauma rTSA). Mean age at time of surgery was 77.3 years for cemented implants and 75.0 years for uncemented implants and demographic and surgical factors were comparable between the 2 groups. Patient demographics by humeral component type are outlined in Table I.

**Table I:** Baseline characteristics of patients undergoing reverse total shoulder arthroplasty for fracture by humeral component type (cemented vs. uncemented).

	N	Minimum	Maximum	Mean	Standard deviation	*P* value
Age (yr)						
Uncemented	517	47.0	96.1	75.0	8.7	.000
Cemented	326	19.8	94.3	77.3	8.8	
BMI						
Uncemented	147	17.0	58.0	30.2	7.6	.032
Cemented	49	15.0	42.0	27.6	5.4	
Follow-up (yr)						
Uncemented	517	0.02	20.4	5.41	3.17	.172
Cemented	326	0.01	18.1	5.73	3.44	

	Uncemented		Cemented		*P* value
	Frequency	Percent	Frequency	Percent	
Gender					
Female	428	82.8%	290	89.0%	.014
Male	89	17.2%	36	11.0%	
Approach					
Deltopectoral	440	85.1%	239	73.3%	<.001
ASA					
1	26	5.1%	15	4.7%	
2	234	46.3%	133	42.1%	
3	228	45.1%	153	48.4%	
4	17	3.4%	15	4.7%	.523

*ASA*, American Society of Anesthesiologists; *BMI*, body mass index.

### Implant survival

**Figure 1:**
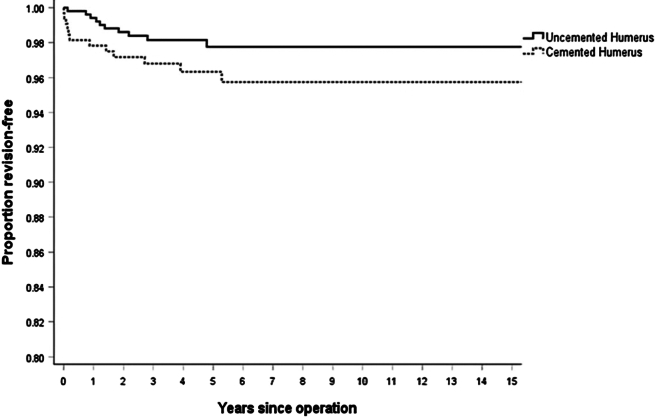
Kaplan-Meier graph demonstrating survival of reverse total shoulder arthroplasty performed for fracture by humeral component type (cemented vs. uncemented).

Revision rates and component year data are outlined in Table II. Kaplan-Meier curves representing implant survival by surgical indication over time are illustrated in Fig. 1. The number of revisions per 100 component-years for cemented trauma rTSA was 0.64, compared to 0.36 for uncemented trauma rTSA. This difference was not statistically significant (*P* = .122).

**Table II:** Revision rate for reverse total shoulder arthroplasty performed for fracture by humeral component type (cemented vs. uncemented).

	All revisions						*P* value	Hazards ratio	Lower 95% CI	Upper 95% CI	Adjusted for age and sex			
	N	Sum comp. Yrs	Revisions	Rate/100-component-years	Lower 95% CI	Upper 95% CI					*P* value	Hazards ratio	Lower 95% CI	Upper 95% CI
Uncemented	517	2,799	10	0.36	0.17	0.66	.122	1.917	0.828	4.438	.029	2.582	1.103	6.043
Cemented	326	1868	12	0.64	0.33	1.12								

*CI*, confidence interval.

### Reason for revision

Reasons for revision are outlined in Table III. For cemented humeral components, the most common cause for revision was dislocation, for uncemented humeral components, the most common cause for revision was deep infection. In the New Zealand Joint Registry, the operating surgeon can select more than 1 ‘indication’ for a revision procedure.

**Table III:** Reason for revision for reverse total shoulder arthroplasty initially performed for fracture by humeral component type (cemented vs. uncemented).

	Uncemented		Cemented	
	Frequency	Percent	Frequency	Percent
Deep infection	3	30.0%	2	16.7%
Glenoid loosening	1	10.0%	0	0.0%
Humeral loosening	1	10.0%	3	25.0%
Dislocation	2	20.0%	6	50.0%
Posterior instability	0	0.0%	1	8.3%
Fracture of humerus	2	20.0%	0	0.0%
Other	2	20.0%	1	8.3%

Multiple indications can be selected for each revision hence the percentages add up to over 100%.

### Patient-reported outcomes

Mean Oxford Scores 6 months postoperatively were 30.4 for cemented trauma rTSA and 31.3 for uncemented trauma rTSA (*P* = .365). Mean Oxford Scores 5 years postoperatively were 36.2 for cemented trauma rTSA and 39.5 and for uncemented trauma rTSA (*P* = .049) (Table IV).

**Table IV:** Oxford Shoulder Scores at 6 mo for reverse total shoulder arthroplasty performed for fracture by humeral component type (cemented vs. uncemented).

	Oxford Score 6 months					*P* value
	N	Minimum	Maximum	Mean	Standard deviation	
Uncemented	280	2	48	31.3	10.1	.365
Cemented	168	7	48	30.4	10.7	

	Oxford Score 5 years					*P* value
	N	Minimum	Maximum	Mean	Standard deviation	
Uncemented	78	11	48	39.5	8.5	.049
Cemented	51	9	48	36.2	10.4	

### Trends over time

**Figure 2:**
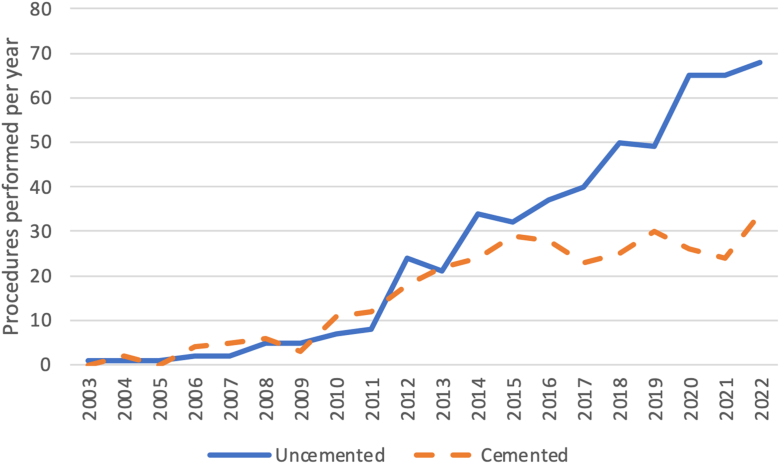
Annual number of uncemented and cemented reverse total shoulder arthroplasty procedures performed for proximal humerus fracture in New Zealand between 2003 and 2022.

Throughout the study period, an increasing proportion of rTSA performed for fracture were using an uncemented humeral component. 43.6% of trauma rTSA procedures between 2002 and 2010 were performed with an uncemented stem compared to 68.4% of procedures performed between 2019 and 2022 (Fig. 2).

## Discussion

Our data suggest there is no statistically significant difference in revision rates between rTSA performed for fracture using a cemented humeral component and rTSA performed for fracture using an uncemented humeral component.

Further studies support this equivalent survival between cemented and uncemented humeral components in the context of proximal humerus fracture. A systematic review by Rossi et al,^21^ which included 1,623 patients across 45 studies, found that at a mean follow-up of 34.6 months, there were no significant differences in reoperation rates between cemented and uncemented rTSA performed for fracture. They did, however, note that the incidence of postoperative all-cause complications was significantly lower in the cemented stem cohort (5.5% vs. 9.7%).^21^ In another review by Phadnis et al,^17^ no difference in the risk of stem loosening or revision was found between cemented and uncemented humeral components in rTSA performed for fracture. Some differences were noted between the groups, however, with uncemented stems having a higher likelihood of early humeral stem migration and nonprogressive radiolucent lines and cemented stems having a higher likelihood of postoperative fractures of the acromion, infection, nerve injury, and thromboembolism.

This equivalent survival between cemented and uncemented stems in the context of fracture may reflect the strengths and limitations of both methods. Metaphyseal fixation is often disrupted in proximal humerus fractures, but modern humeral components may have satisfactory diaphyseal fixation alone.^7^^,^^16^^,^^20^ Stem geometry is therefore a key consideration in implant selection and use of cement. Furthermore, while cement may have advantages in osteoporotic bone, it brings with it potential further issues including increased operative time, cement disease, and further considerations in revision surgery.^2^^,^^7^^,^^25^^,^^26^ Another consideration is the specific implant chosen as all humeral implants, both cemented and uncemented, differ in their designs. The majority of rTSA procedures in New Zealand utilize either the Systema Multiplana Randelli (SMR; Lima-LTO, Villanova Italy) or the Delta Xtend (DePuy Synthes, Raynham, MA, USA)^27^ and it is possible that certain implant characteristics, such as the diaphyseal fit of the SMR, make it more appropriate in the context of fracture.

Reason for revision was also analyzed in the current study, with the most common reason for revision in the cemented group being dislocation, and the most common reason for revision in the uncemented group being deep infection (Table III). It is unclear what the underlying reason for this might be, and due to the relatively small numbers of revision, it is difficult to comment on the importance of this finding.

The OSSs observed in our study are broadly in keeping with previous studies of OSSs following rTSA. Chaudhury et al reported mean OSSs of 38 for uncemented rTSA at 2 years postoperatively in a United Kingdom cohort,^4^ while Youn et al in an analysis of 20 patients found a mean OSS of 42.5.^27^ In the current study, there were no statistically significant differences in OSSs at 6 months postoperatively. At 5 years, uncemented stems had statistically significantly greater mean Oxford Scores (39.5) compared to cemented stems (36.2) (*P* = .049), although this difference is less than the minimally clinically important difference reported by Nyring et al^15^ and Jones et al^10^ In keeping with this, a systematic review by Rossi et al,^21^ found no significant differences in range of motion (ROM), visual analog scale scores, Constant-Murley scores, or rate of tuberosity healing between cemented and uncemented rTSA performed for fracture, although they did find that the mean postoperative American Shoulder and Elbow Surgeons (ASES) score was statistically significantly lower in the cemented cohort than in the uncemented cohort. In contrast, however, a review by Kao et al found that patients who received a cemented rTSA for proximal humerus fracture had significantly higher Constant-Murley scores than for patients who received an uncemented rTSA for the same indication,^12^ and review by Phadnis et al^17^ found functional outcome and range of movement were equivalent between cemented and uncemented rTSA for proximal humerus fracture. At this stage, there appears to be no functional outcome difference between the treatment options.

Functional outcomes following rTSA for trauma depend on tuberosity healing, rather than cemented or uncemented humeral components. In a radiographic and clinical study of 67 patients, Rossi et al found at a mean follow-up of 41 months that there was no significant differences in postoperative ROM, functional outcomes (the visual analog scale for pain, the ASES score, the Constant score, and Single Assessment Numeric Evaluation score) or failure rates between patients with cemented rTSA and uncemented rTSA for proximal humeral fractures.^20^ They did find, however, on radiographic analysis, that patients with healed tuberosities had significantly better ROM and functional scores than those without healed tuberosities.^20^ While there is some evidence that tuberosity healing rates are higher with use of uncemented stems,^11^ the review by Rossi et al,^21^ showed no different in the rate of tuberosity healing between cemented and uncemented rTSA performed for fracture.

Another key consideration is the fact that surgeons must be familiar with implants in order to achieve optimal outcomes. Our findings demonstrate no advantage of cemented stems over uncemented stems for proximal humerus fracture, but this is in the context of rising use of uncemented humeral components in New Zealand^27^ and growing and notably more frequent use of uncemented stems for proximal humerus fractures than cemented stems (Fig. 2). In contrast, a registry study of rTSA for all indications from Norway, where the vast majority of humeral components are cemented, found that Delta III (uncemented stem) had a higher risk of revision compared with Delta Xtend (cemented stem).^8^ This may represent the significant influence surgeon experience can have on surgical outcomes.

These findings are in the context of rTSA becoming increasingly important in the management of proximal humerus fractures. In an analysis of Australian national health-care databases, McLean et al found a significant increase in the incidence of proximal humerus fractures between 2008 and 2017, and that use of rTSA for these injuries has increased significantly in the same period from 4.1% to 24.5% of surgically managed fractures. This was despite a decrease in overall operative management in the same period, and coincided with a significant reduction in hemiarthroplasty.^14^ With increasing use of rTSA in proximal humerus fractures, we have an increasing body of data upon which to base decisions, and reassuringly, our findings are in keeping with the most recent evidence. A 2025 study by Raubenheimer et al^19^ investigated revision rates and mortality following cemented and uncemented rTSA for proximal humerus fracture, and found that revision rates and mortality were comparable between the groups. Furthermore, a further analysis of a subset of patients at a single institution was analyzed for ASES score, OSSs and single assessment numerical value, with no significant differences between the groups.

This study has several limitations. Firstly, cemented and uncemented stems represent a large number of different implants that will have different longevities, although it must be noted that the majority of rTSA procedures performed in New Zealand utilize either the SMR or Delta Xtend.^27^ Secondly, the NZJR will not capture early minor complications that do not result in reoperation, revision or mortality, and no radiographic analysis was performed. Thirdly, the number of revision procedures included in this study means that reason for revision cannot be meaningfully analyzed. Radiological outcomes were also not analyzed. Finally, given this was a retrospective study, cementing was not randomized and was used in different patient populations. To account for baseline differences, a multivariate Cox proportional hazards regression model was used to compare revision risk, however this may not account for more qualitative or radiographic differences between the 2 groups, for example, an assessment of bone stock by an experienced surgeon or fracture morphology.

## Conclusion

In the context of increasing use of rTSA for acute proximal humerus fractures, revision rates and patient-reported outcomes are similar between cemented and uncemented humeral stems. For certain patients, uncemented humeral components may therefore be an acceptable first-line treatment.

## Disclaimers:

Funding: No funding was disclosed by the authors.

Conflicts of interest: The authors, their immediate families, and any research foundation with which they are affiliated have not received any financial payments or other benefits from any commercial entity related to the subject of this article.
